# Supplementary material for: Geometric Wheat Modeling and Quantitative Plant Architecture Analysis Using Three-Dimensional Phytomers
Source: Plants (Basel). 2023 Jan 18;12(3):445. doi: 10.3390/plants12030445 (PMC9919470; doi:10.3390/plants12030445)
Supplement: Supplementary file 1 [file plants-12-00445-s001.zip › Supplementary_Table_S1.pdf]

Table S1. Detailed parameter definition and description of 3D phytomere of wheat.

Classification	Abbreviation	Parameter	Description
Whole phytomer	$h_i^{Phytomer}$	Phytomer height	Vertical distance from the lowest point of the phytomer to ground.
	$\alpha_i^{Phytomer}$	Phytomer azimuth	
Leaf	$h_i^{LeafBase}$	Leaf base height	Vertical distance from leaf base to ground
	$h_i^{LeafTop}$	Leaf top height	Vertical distance from the highest point to ground
	$h_i^{LeafTip}$	Leaf tip height	Vertical distance from leaf tip to ground
	l_i^{Leaf}	Leaf length	
	w_i^{Leaf}	Leaf width	
	θ_i^{Leaf}	Leaf angle	
	α_i^{Leaf}	Leaf azimuth	
	s_i^{Leaf}	Leaf area	
Sheath	$h_i^{SheathBase}$	Sheath base height	Vertical distance from sheath base to ground
	l_i^{Sheath}	Sheath length	
	θ_i^{Sheath}	Sheath angle	Sheath inclination
	$d_i^{SheathMax}$	Sheath maximum diameter	
	$d_i^{SheathMin}$	Sheath minimum diameter	
Internode	$h_i^{InternodeBase}$	Internode base height	$h_i^{InternodeBase} = h_i^{SheathBase}$。
	$l_i^{Internode}$	Internode length	
	$\theta_i^{Internode}$	Internode angle	Internode inclination
	$d_i^{InternodeMax}$	Internode maximum diameter	
	$d_i^{InternodeMin}$	Internode minimum diameter	
Appendage	l_i^{Spike}	Spike length	
	θ_i^{Spike}	Spike bearing angle	
	$d_i^{NodeMax}$	Node maximum diameter	
	$d_i^{NodeMin}$	Node minimum diameter	
	$n_i^{NodalRoot}$	Nodal root number	
	$d_i^{NodalRoot}$	Averaged diameter of nodal roots	
